# Changes in Arterial Blood Pressure and Oxygen Tension as a Result of Hoisting in Isoflurane Anesthetized Healthy Adult Horses

**DOI:** 10.3389/fvets.2020.601326

**Published:** 2020-11-25

**Authors:** Michelle Cerullo, Bernd Driessen, Hope Douglas, Klaus Hopster

**Affiliations:** Department of Clinical Sciences, University of Pennsylvania, School of Veterinary Medicine, Kennett Square, PA, United States

**Keywords:** horse, anesthesia, blood pressure, arterial oxygenation, hoisting

## Abstract

**Background:** In anesthetized adult horses, changes in recumbency can influence the cardiovascular system but how arterial blood pressures and oxygen tension change in isoflurane anesthetized animals as a direct result of hoisting has not been investigated.

**Objective:** To evaluate effects of hoisting on hemodynamic function and pulmonary gas exchange in isoflurane-anesthetized horses.

**Study Design:** Prospective, experimental study.

**Methods:** Six adult horses were anesthetized three times using isoflurane in pure oxygen (inspired fraction 0.9–1.0), and allowed breathing spontaneously in lateral recumbency. After 45 min horses were hoisted using a single hoist-hobble system for 5 min and returned into left lateral recumbency. Heart rate (HR), respiratory rate (RR), and systolic (SAP), diastolic (DAP), and mean arterial blood pressures (MAP) were measured every minute starting from 5 min before to 5 min after hoisting. Arterial blood gas samples were collected before, during, and after hoisting.

**Results:** Significant changes in hemodynamic parameters and PaO_2_ but not PaCO_2_ were found between baseline recordings and measurements obtained during and early after hoisting. The MAP decreased within the 1st min of hoisting from a mean of 74 ± 17 mmHg at baseline to 57 ± 20 mmHg (*p* < 0.05). Thereafter, it rapidly recovered to baseline before continuing to rise to higher than baseline values and then remaining elevated for 5 min after horses were returned into lateral recumbency. Simultaneously, the HR increased by 6–9 beats per min during the initial 3 min of hoisting before returning close to baseline values (*p* < 0.05). The PaO_2_ decreased significantly from a mean of 324.9 ±137.0 mmHg at baseline to a mean of 141.3 ± 104.2 mmHg during hoisting (*p* < 0.001) without recovering any more to baseline values.

**Clinical significance:** Hoisting an adult horse during or at the end of isoflurane anesthesia carries the risk of a precipitous, though short-lived (1–2 min), drop in arterial blood pressures and a persistent decrease in arterial oxygenation. While in systemically healthy animals the observed functional impairments were not life-threatening, they may be more severe in systemically compromised horses.Therefore, arterial blood pressures and oxygenation must be carefully monitored when hoisting sick equine patients during or at the end of inhalant anesthesia.

## Introduction

Risk factors contributing to the relatively high equine morbidity and mortality rate associated with general anesthesia have been extensively investigated over the last several decades. A multitude of risk factors have been reported including the relative contribution of hypotension and hypoxemia both intraoperatively and during recovery (1, 2). Volatile anesthetics and patient position adversely affect cardiovascular and respiratory systems (3, 4). Equine patients are especially susceptible to oxygenation impairment under anesthesia as a result of marked ventilation/perfusion (V/Q) mismatching secondary to increased pulmonary atelectasis formation and intrapulmonary shunting (5). Cardiovascular parameters including arterial blood pressure and cardiac output of spontaneously breathing anesthetized ponies have been shown to be affected by body positioning, with the most pronounced decrease of those parameters occurring with animals in dorsal recumbency (6). Further, a decrease in PaO_2_ and increase in the P_A−a_O_2_ gradient, encountered in anesthetized horses (7, 8) is well-documented and likely caused more by recumbency than the anesthetic agents used (9), although anesthetics may influence the magnitude of those changes. Post-anesthetic hypoxemia has been documented in the recovery period even in horses that had shown only mild arterial oxygenation impairments during anesthesia (10–12).

To the best knowledge of the authors, there has only been one investigation into the effects of hoisting on arterial blood pressures and oxygenation in anesthetized horses. Braun et al. studied cardiovascular and oxygenation changes in foals under total intravenous anesthesia (TIVA) before and after hoisting to the ipsilateral or contralateral recumbency (13). The study revealed a significant decrease in oxygenation; however, no significant difference in cardiovascular parameters was evident after hoisting between foals placed in the same or contralateral recumbency (13).

Many equine hospitals and private practice surgical facilities utilize a hobble-hoist system to facilitate movement and positioning of equine patients after induction of anesthesia on a surgery table, and—after surgery—back onto the floor of the recovery stall. However, to the authors' knowledge, there have been no studies determining in adult horses the effects of hoisting on cardiovascular and respiratory system function.

The objective of this study was to investigate effects of hoisting on hemodynamic function and pulmonary gas exchange in isoflurane-anesthetized horses. Based on previous clinical observations, we hypothesized that hoisting will cause primarily a significant decrease in arterial blood pressures and oxygen tension (P_a_O_2_) and that those changes will continue into the period following hoisting.

## Materials and Methods

### Horses

Six horses including three Standardbreds, one Warmblood Horse, one Quarter Horse, and one Thoroughbred cross (three mares, three geldings) with a mean age of 11 (6–17) years and body weight of 503 (339–606) kg were included in this study. These horses showed no signs of cardiovascular or pulmonary abnormalities based on history and physical exam. All horses were anesthetized being part of a separate study evaluating drug reversal and recovery quality. The study was approved by the Institutional Animal Care and Use Committee, approval number 806641-aaedbaj.

### Study Design

Heart rate (HR), arterial blood pressures, and end-tidal isoflurane concentration were monitored every 5 min. A 20-g catheter was inserted into the facial artery for continuous invasive blood pressure measurement and blood sampling. The pressure transducer was zeroed against atmospheric pressure and sutured to the horse at the level of the thoracic inlet while in lateral recumbency. The duration of anesthesia was set to 90 min based on the main study protocol.

### Experimental Protocol

A 7x7 cm area over the left jugular vein was clipped and aseptically prepared for catheter placement. Local anesthetic, 2 mL of lidocaine (Lidocaine Hydrochloride 2%) was infiltrated subcutaneously and a 14-gauge catheter was placed percutaneously. Horses were sedated using 0.8 mg/kg b.w.t. xylazine (X-Ject E® 100 mg/mL), and anesthesia was induced with 2.2 mg/kg b.w.t. ketamine (Ketathesia® 100 mg/mL) and 0.05 mg/kg b.w.t. midazolam (Midazolam 5 mg/mL) i.v. Each horse was placed in left lateral recumbency on a recovery stall mat. Intubation was performed using a 20- or 22-mm diameter tube (JorVet Silicone) nasotracheally and horses were allowed to breath spontaneously. The oxygen was selected to be administered at 100% on the Tafonius. Anesthesia was maintained with isoflurane (Isoflurane, USP) targeting an end-tidal concentration of 1.1 vol%. Each horse was anesthetized and underwent the experiment three times with a washout period of at least 2 weeks between anesthetic events.

### Data Collection

**Figure 1:**
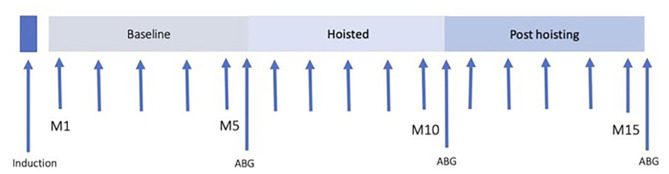
Time line of experimental protocol. Forty-five minutes after induction, HR, SAP, MAP, and DAP were recorded. Immediately prior to hoisting and after 5 min of hoisting, and 5 min after return to lateral recumbency, arterial blood gas samples were drawn and instantly analyzed.

After induction of anesthesia, horses were instrumented and allowed to stabilize under anesthesia. Forty-five minutes after induction of anesthesia HR, systolic (SAP), diastolic (DAP), and mean arterial pressure (MAP), and respiratory rate (RR) were recorded every minute for 5 min and an arterial blood sample was taken immediately prior to hoisting. Horses were then suspended in dorsal using a hobble-ring-hoist system with the hobbles placed above the fetlocks of all four limbs and one additional hoist to support the head at the level of the heart. Horses were hoisted for 5 min while continuing to breathe the same gas mixture, and parameter recording was continued at 1-min intervals. After 5 min, another arterial blood sample was collected immediately before placing each horse back into left lateral recumbency on the mat. All parameters were monitored every minute for another 5 min followed by a post-hoisting arterial blood sample collection for analysis (Figure 1).

### Blood Gas Analysis

Arterial blood samples were drawn using Line Draw Arterial Blood Sample Syringes (Smith Medical) and immediately analyzed for PaO_2_ and carbon dioxide tension (PaCO_2_) (OptiMedical). The blood gas analyzer was calibrated with reference gases of 6% CO_2_, 15% O_2_, balanced N_2_ at 28 psi and 21°C (OptiMedical Calibration Gas) for accuracy.

The O_2_ content-based index (F-shunt) was calculated as described by Araos et al. (14):

([Cc‘O_2_ – CaO_2_]/[Cc‘O_2_ – CaO_2_ + C(a – v)O_2_]) × 100

where 3.5 mL/dL is a fixed value of C(a – v) O_2_

and CaO_2_ = Hb × 1.31 × SaO_2_ + 0.003 × PaO_2_

and Cc‘O_2_ = Hb × 1.31 × Sc‘cO_2_ + 0.003 × Pc‘O_2_

with Hb being venous hemoglobin concentration (g/dL), 1.31 the oxygen-carrying capacity of hemoglobin (mL/g), SO_2_ the oxygen saturation, 0.003 the solubility coefficient of oxygen in plasma, and PO_2_ the partial pressure of oxygen (mm Hg).

with Pc‘O_2_ = P_A_O_2_ = FiO_2_ × (Pb – PH_2_O) – (PaCO_2_^*^1.2)

where Pb is barometric pressure (mmHg), PH_2_O is vapor pressure of water (mmHg), and 1.2 is 1/respiratory quotient. The barometric pressure was recorded by https://www.timeanddate.com/weather/usa/philadelphia/historic.

### Data Analysis

A total of 18 experiments were performed in six animals. For HR and arterial blood pressures all data of the five initial measurements before hoisting were pooled and presented as one baseline data point in Table 1 for each parameter since the means of those values collected for each minute in the 5 min preceding the hoisting event did not differ significantly from each other. All data were tested for normality using visual assessment of the OO plots and Shapiro-Wilk test.

**Table 1:** Mean and *SD* of the parameters heart rate (HR), systolic (SAP), mean (MAP), diastolic arterial pressure (DAP), respiratory rate (RR), arterial partial pressures of oxygen (PaO_2_), carbon dioxide (PaCO_2_), and F-shunt in horses prior to, during, and after hoisting with return into lateral recumbency.

	**HR in 1/min**	**SAP in mmHg**	**MAP in mmHg**	**DAP in mmHg**	**RR in 1/min**	**PaO_**2**_ in mmHg**	**PaCO_**2**_ in mmHg**	**F-shunt in %**
Baseline	36 ± 4	104 ± 21	74 ± 17	59 ± 17	5 ± 3	324.9 ± 136.0	62.8 ± 12.4	22 ± 2.4
M6	41 ± 8^*^	83 ± 26^*^	57 ± 20 ^*^	40 ± 19^*^	6 ± 2			
M7	44 ± 11^*^	104 ± 32	82 ± 23	69 ± 20	5 ± 3			
M8	43 ± 15^*^	136 ± 46	104 ± 28^*^	88 ± 27^*^	7 ± 2			
M9	40 ± 8	149 ± 44^*^	117 ± 32^*^	92 ± 32^*^	7 ± 3			
M10	41 ± 9	154 ± 51^*^	124 ± 32^*^	103 ± 24^*^	7 ± 4	141.3 ± 104.2^*^	48.6 ± 10.7	31 ±2.1^*^
M11	44 ± 13^*^	160 ± 37^*^	126 ± 29^*^	106 ± 28^*^	9 ± 4			
M12	31 ± 4	150 ± 29^*^	115 ± 20^*^	95 ± 21^*^	8 ± 6			
M13	29 ± 3	138 ± 24^*^	104 ± 16^*^	87 ± 17^*^	5 ± 3			
M14	30 ± 4	136 ± 20^*^	101 ± 15^*^	87 ± 15^*^	6 ± 4			
M15	32 ± 4	131 ± 19^*^	99 ± 15^*^	84 ± 15^*^	6 ± 4	172.4 ± 108.4 ^*^	62.7 ± 9.8	29 ± 2.2

* *Indicates statistically difference from baseline (p < 0.05)*.

*Baseline = M1–M5 period with horses in lateral recumbency before hoisting*.

*M6–M10 = period of hoisting*.

*iM11–M15 = period with horses in lateral recumbency after hoisting*.

Statistical analysis was performed by repeated measures analysis of variance. The analysis included a *post-hoc t-test* for comparison of individual measurements. Bonferroni correction was performed if appropriate. The level of significance was set at 5%, *p* < 0.05. Data are presented as means ± standard deviation (*SD*).

## Results

### Heart Rate

The HR increased significantly (*p* < 0.001) during hoisting, rising rapidly from baseline 35 ± 4 beats/min to 44 ± 11 beats/min and then staying elevated before returning back to baseline 1 min after positioning the horse back into lateral recumbency (Table 1).

### Arterial Blood Pressure

**Figure 2:**
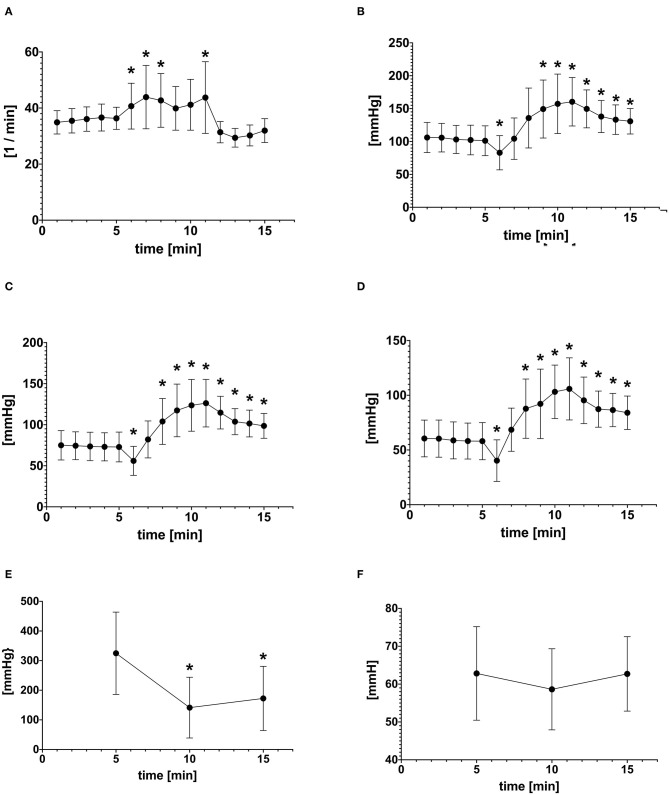
Mean ± *SD* of **(A)** Heart rate (HR) **(B)** Systolic arterial pressure (SAP) **(C)** Mean arterial pressure (MAP) **(D)** Diastolic arterial pressure (DAP) at each time point before (1–5), during (6–10), and after hoisting (11–15) **(E)** PaO_2_ and **(F)** PaCO_2_ before (M5), during (M10), after (M15) hoisting. *Indicates statistically difference from baseline (*P* < 0.05).

Hoisting the anesthetized animals resulted in an immediate decrease in SAP, MAP, and DAP from baseline within the 1st min of hoisting (M6) (Figure 2). The arterial blood pressures returned back to baseline level within the subsequent minute and continued rising, even beyond baseline values (Figure 2). The arterial blood pressures remained elevated for 5 min after horses were laid back into lateral recumbency (Figure 2). In 3/18 experiments, the arterial catheter was mildly compressed by the halter during hoisting potentially dampening arterial blood pressure values during M6–M10. In these three experiments the MAP was not significantly different when compared to the other 15 experiments. Therefore, these three experiments remained included in the analysis.

### Respiratory Rate

While the respiratory rate did not change significantly from baseline, there were changes noted in that the mean RR increased slightly from 5 ± 3 breaths/min at baseline to 7 ± 3 breaths/min toward the end of hoisting (Table 1). Likewise, during the 1st min of returning to lateral recumbency the RR increased to 9 ± 4 breaths/min and gradually returned to near baseline with 6 ± 4 breaths/min.

### Arterial Blood Gases

The PaO_2_ decreased significantly from 324 mmHg at baseline to 141 mmHg during hoisting (*p* < 0.001; Table 1). Five minutes after hoisting the PaO_2_ still had not changed significantly from data obtained during hoisting (*p* = 0.256; Table 1). There were no significant changes in PaCO_2_ observed during or after hoisting. The shunt fraction (Table 1) increased significantly from baseline after hoisting and decreased after returning to lateral recumbency.

## Discussion

In this study, dynamic and significant changes to both cardiovascular and respiratory parameters were noted during and after hoisting. Heart rate and arterial blood pressures changed markedly from baseline during hoisting.

An initial period of hypotension with an increase in HR immediately after hoisting was consistently followed by a rapid and progressive increase in arterial blood pressures. The results of this study revealed an initial mixed systolic/diastolic hypotension immediately after hoisting with a return to baseline within 1 min. Changes in DAP are most commonly due to a change in the conducting vessels' resistance (i.e., vasomotor tone) under isoflurane anesthesia (15). Changing position from lateral to dorsal recumbency during hoisting may initially exacerbate hypotension secondary to shifting weight of abdominal organs onto the caudal vena cava, thereby decreasing venous return to the heart. After the initial decrease in arterial blood pressures, the rapid increase observed was likely due to a central redistribution of peripheral blood because of a change in gravitational forces, increase in preload, and increase in arterial pressure secondary to the Frank-Starling mechanism (16). Horses under halothane anesthesia displayed cardiovascular changes associated with positional changes. The greatest decrease in cardiovascular system function in both spontaneously and mechanically ventilated ponies can be seen in dorsal recumbency with a decrease in myocardial contractility while maintaining systemic arterial blood pressures (6). Total peripheral resistance has been shown to change insignificantly in lateral recumbency and to increase with horses in dorsal recumbency, which may be the result of compensatory vasoconstriction in this position (6).

In addition to the physiologic response, horses were maintained in a light plane of anesthesia before hoisting with an end-tidal isoflurane of ~1.1%, which is below the reported MAC of 1.3% for the equine (17). The tension hobbles exert on the underlying musculoskeletal structures during hoisting is likely stimulating nociceptors. The slip rope design of the hobble may cause a tourniquet effect, which has been shown to lead to hypertension in anesthetized horses (18, 19). In this population of horses, 5/6 horses developed hypertension for at least 1 min during hoisting. Although horses in this study were maintained on isoflurane/oxygen while being hoisted, some horses developed slow nystagmus and one horse exhibited slight movement. Therefore, it could be considered that horses with orthopedic or musculoskeletal pathology such as arthritis could experience an increase in noxious stimulation. Further investigation is warranted to clarify the role of musculoskeletal pathology in stimulation during hoisting. Cardiovascular parameters recorded in this study were limited to HR and arterial blood pressures. Cardiac output and pulmonary artery pressure measurements during hoisting would have provided further information about pulmonary perfusion changes during hoisted flexion as well changes to global perfusion when considering more critically sick patients.

Oxygen tension decreased significantly during hoisting and remained below baseline when horses returned back to left lateral recumbency. Horses are commonly subject to a marked V/Q mismatching under inhalant anesthesia due to changes in lung volume and function when placed in different recumbences and due to interference of volatile anesthetics with the hypoxic pulmonary vasoconstriction response. Reduced oxygen tensions in anesthetized horses has been documented in both laterally recumbent and dorsally recumbent horses with a larger alveolar-arterial (A-a) gradient observed in dorsal recumbency (5, 20–24). The decrease in functional residual capacity and atelectasis formation are most pronounced in dorsal recumbency as abdominal organs shift cranially to push against the diaphragm and thus compress a larger portion of both lungs leading to an increase in collapsing alveolar units (25). Equine lungs have minor collateral ventilation; however, not enough to contribute to ventilation of associated alveoli and therefore this mechanism does not contribute to improved oxygenation as seen in other species (26). In this study, the FiO_2_ remained at least at ≥ 90% for at least 40 min before the first ABG analysis was performed, which is enough time for significant denitrogenation to occur which promotes formation of absorption atelectasis. A single hoist system necessarily pulls fore and hind limbs together to one central point, thereby increasing intra-abdominal as well as transpulmonary pressures, which likely exacerbates compression atelectasis and hence further impairs gas exchange. Spinal flexion posture, such as occurs during hoisting, has been shown to significantly decrease maximal inspiratory and expiratory pressures, as well as decreased respiratory muscle strength compared to the upright posture when investigated in adults (27). When suspending spontaneously breathing horses, the additive effect of compression atelectasis, decreased functional residual capacity, and increased respiratory muscle effort required to expand the thoracic cavity against abdominal pressure appears to lead to a significant decrease in PaO_2_.

Increased pulmonary shunting has also been observed during anesthesia involving a higher fraction of inspired oxygen (FiO_2_) anesthesia (23, 28, 29). As the FiO_2_ did not change during the experiment, the contribution of high FiO_2_ induced intrapulmonary shunting would have been minimal. Furthermore, previous studies failed to show an improvement in alveolar dead space ventilation or shunt fraction in dorsally recumbent horses by reducing the FiO_2_ to 0.5 (30). The shunt fraction increased significantly from baseline solely after hoisting and decreased mildly once returned to lateral recumbency. Arterial oxygenation in horses can worsen over time but usually stays in acceptable limits if conditions do not change (31). In mechanically ventilated horses positioned in dorsal recumbency, PaO_2_ measured over time does not significantly decrease from baseline. Therefore, we assume that the drop in PaO_2_ was related to the change in positioning and not related to any time effect.

The mean PaCO_2_ pre-hoisting was 62 mmHg indicating mild to moderate hypoventilation during baseline spontaneous ventilation. While this is another common cause of decreased PaO_2_ that can be treated by increasing the FiO_2_, it is unlikely the main contributing factor (32). In this study, adequate ventilation during hoisting was indicated by the decrease in PaCO_2_ and mild increase in respiratory rate, though neither parameter changed statistically significantly.

This study was performed under controlled experimental conditions, however there were several limitations that should be recognized. Although arterial catheter placement was successful in each horse, placement of the catheter varied between experiments. Catheters were initially placed in the transverse facial artery adjacent to the halter strap during the first three experimental procedures. During hoisting, the pressure exerted by the halter may have compressed the artery and excessively dampened the arterial pressure wave resulting in lower than accurate systolic, mean, and diastolic pressure readings for those 5 min. Although these catheters subjectively seemed compressed and the waveform dampened, they remained patent, a distinguished arterial waveform was displayed on the monitor, and arterial pressures increased at a similar rate and magnitude compared to the situation of catheters that were not under pressure of the halter. During subsequent experiments, the arterial catheter was positioned to be away from the pressure points of the halter. The horses anesthetized in this study were underwent a single hoisting. In a clinical setting, horses are hoisted onto and off of a surgical table and therefore might lead to potential worsened oxygenation during the second hoist. All horses in this experiment continued to spontaneously breathe an oxygen rich gas mixture during and after hoisting. Although no horse was considered hypoxemic based on blood gas analysis data, in practice, horses are not commonly provided oxygen supplementation during hoisting. Interpretation of the changes of PaO_2_ should take this into consideration and horses may become hypoxemic during those times.

## Conclusion

Despite the administration of pure oxygen, hoisting an adult horse during or at the end of isoflurane anesthesia carries the risk of a precipitous, though short-lived (1–2 min), drop in arterial blood pressures and a persistent decrease in arterial oxygenation. While in systemically healthy animals the observed functional impairments were not life threatening, they may be more severe and longer lasting in systemically compromised horses and therefore much attention must be paid to monitoring arterial blood pressures and oxygenation when hoisting sick equine patients.

## Data Availability Statement

The raw data supporting the conclusions of this article will be made available by the authors, without undue reservation.

## Ethics Statement

The animal study was reviewed and approved by International Animal Care and Use Committee.

## Author Contributions

MC was responsible for data collection, interpretation, manuscript writing, and submission. BD was responsible for overseeing experimental protocol, data analysis, and manuscript editing. HD was responsible for data acquisition. KH was responsible for conception of study, supervision of collection, data analysis, and manuscript editing. All authors contributed to the article and approved the submitted version.

## Conflict of Interest

The authors declare that the research was conducted in the absence of any commercial or financial relationships that could be construed as a potential conflict of interest.
